# Grouping of genomic markers in populations with family structure

**DOI:** 10.1186/s12859-021-04010-0

**Published:** 2021-02-19

**Authors:** Dörte Wittenburg, Michael Doschoris, Jan Klosa

**Affiliations:** Institute of Genetics and Biometry, Leibniz Institute for Farm Animal Biology, 18196 Dummerstorf, Germany

**Keywords:** Single nucleotide polymorphism, Covariance matrix, Clustering, TagSNP, Group lasso, SNP-BLUP

## Abstract

**Background:**

Linkage and linkage disequilibrium (LD) between genome regions cause dependencies among genomic markers. Due to family stratification in populations with non-random mating in livestock or crop, the standard measures of population LD such as $r^2$ may be biased. Grouping of markers according to their interdependence needs to account for the actual population structure in order to allow proper inference in genome-based evaluations.

**Results:**

Given a matrix reflecting the strength of association between markers, groups are built successively using a greedy algorithm; largest groups are built at first. As an option, a representative marker is selected for each group. We provide an implementation of the grouping approach as a new function to the R package hscovar. This package enables the calculation of the theoretical covariance between biallelic markers for half- or full-sib families and the derivation of representative markers. In case studies, we have shown that the number of groups comprising dependent markers was smaller and representative SNPs were spread more uniformly over the investigated chromosome region when the family stratification was respected compared to a population-LD approach. In a simulation study, we observed that sensitivity and specificity of a genome-based association study improved if selection of representative markers took family structure into account.

**Conclusions:**

Chromosome segments which frequently recombine in the underlying population can be identified from the matrix of pairwise dependence between markers. Representative markers can be exploited, for instance, for dimension reduction prior to a genome-based association study or the grouping structure itself can be employed in a grouped penalization approach.

## Background

Genomic markers are an invaluable source for characterizing genetic variety and to elucidate the relationship between genetic and phenotypic variation in breeding populations. Dependencies among genomic markers are caused by linkage and linkage disequilibrium (LD) between genome regions. Though this condition complicates investigations on which genetic variants are truly associated with trait expression [1], dependencies can be advantageous for grouping of markers. For example, clustering based on a greedy algorithm [2], hierarchical clustering (e.g., [3]) or grouping via interval-graph modeling [4] exploit the presence of LD blocks which are regions of particularly high correlation. To allow for proper inferences of such approaches, a suitable measure for the strength of dependence is needed. For instance, measuring LD in terms of $r^2$ [5] is a natural choice but it is meaningful only for populations without stratification. In livestock and crop breeding, however, populations are often characterized by strong family stratification due to non-random mating of selected individuals. As examples, large paternal half-sib families are typical for cattle populations whereas chicken or fish populations consist of full-sib families. In plant breeding, maternal half-sib families are often produced in, for instance, wheat and clover. Then, linkage between markers within family leads to haplotype frequencies among progeny that are not conclusive for estimating $r^2$. Hence, there is need to promote measures of marker dependence which takes into account the particular family structure.

Especially in situations of ultra-dense panels of single nucleotide polymorphisms (SNPs), it is often sufficient to investigate representative SNPs (“tagSNPs”) out of each cluster. This subset can help identifying trait-associated genome regions in genome-wide association studies and allows comparing genome characteristics between ethnics/species/breeds (e.g., [2]). As the choice of tagSNPs is a consequence of grouping, it is also influenced by the underlying population structure.

The objective of this paper is to exploit the family structure of a population for specifying groups of associated markers. We generalize the grouping approach of Carlson et al. [2] in order to allow binning of markers given a correlation matrix or any kind of similarity matrix with scaled entries in [0, 1]. We investigate three case studies and a simulation study. For each case study, we visually inspect the correlation matrix and link to the outcome of grouping. Usability for genome-based association studies is shown as one possible field of application. Results were compared to the commonly used population-LD approach which ignores family structure. We provide a new function to the R package hscovar (available at CRAN) that enables grouping of markers and selection of representative markers.

## Methods

The dependence between pairs of SNPs, each with two alleles A and B, can be expressed in terms of a covariance or correlation matrix. It has already been shown in the literature how to calculate the theoretical covariance between markers in a population consisting of half-sib families [1]. It requires a genetic map, haplotypes of the common parent and LD information (or haplotype frequencies) of the population the individual parent comes from. This approach can be extended to be applicable to full-sib families by adding the paternal and maternal contribution into a single covariance matrix; the derivation is summarized in Additional file 1. Hence, a covariance matrix can be derived for any family structure, and this constitutes the input of the following grouping approach.

### Grouping of markers

We generalized the strategy of Carlson et al. [2] for binning markers and selecting representatives to be applicable to any symmetric matrix which reflects a measure of dependence between markers and has entries scaled in [0, 1]. In particular, we considered the correlation matrix *R*. The idea is that SNPs which are associated to each other are assigned to groups. Groups are built one after the other—the largest group at first. For $b=1,2,\ldots$, the *b*-th group is identified by searching for the SNP that has most occurrences of absolute correlation to other SNPs larger than a given threshold *t*. More precisely, let ${\mathcal {S}}_b$ denote the set of SNP indices which have not been binned yet. Then, for each SNP $k\in {\mathcal {S}}_b$, the set of highly associated SNPs is determined as (Step A)

$$\begin{aligned} {\mathcal {C}}_k&= \{l|\, l \in {\mathcal {S}}_b : |R_{k,l}|>t\}, \end{aligned}$$

and a set with highest cardinality (operator #) is chosen,

$$\begin{aligned} c \in \underset{k}{\arg \max } \#{\mathcal {C}}_k. \end{aligned}$$

Thus, ${\mathcal {C}}_c$ constitutes the *b*-th group, and a tagSNP is selected from this group. SNP *c* has strong correlation with any other SNP in ${\mathcal {C}}_c$ but it can happen that also other SNPs of ${\mathcal {C}}_c$ fulfill this criterion. Hence, a set of candidates is given by (Step B)

$$\begin{aligned} {\mathcal {T}}&= \{k |\, k\in {\mathcal {C}}_{c}\wedge \forall l\in {\mathcal {C}}_{c}: |R_{k,l}| > t\}. \end{aligned}$$

**Fig. 1:**
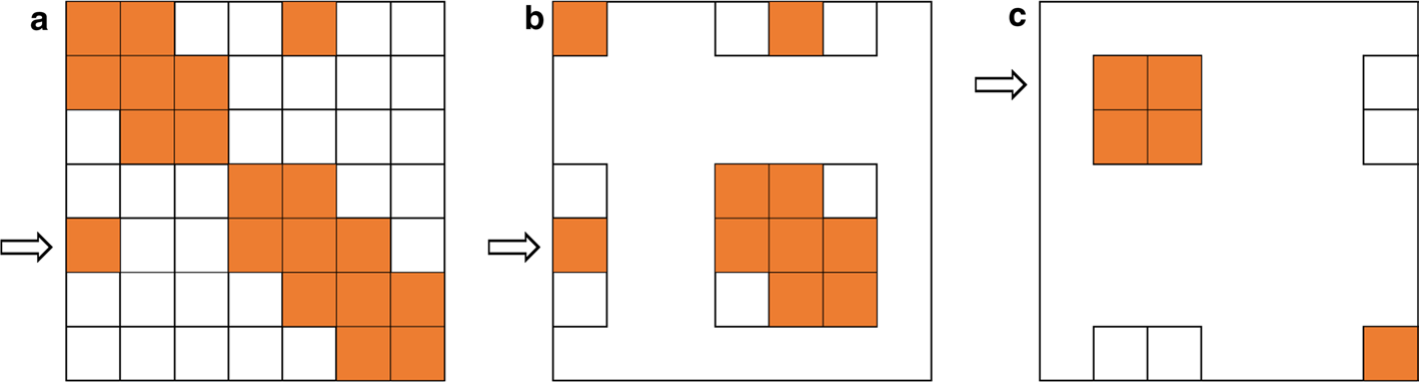
**a** Matrix highlights the SNP pairs with correlation larger than a certain threshold for a given SNP panel. The SNP involved most is marked by an arrow (Step A). **b** Correlations are considered within the corresponding SNP subset and a tagSNP is selected (Step B). **c** All SNPs associated with the tagSNP have been removed from the remaining ungrouped SNP panel and the next round of iteration starts with Step A. Iterations continue until no ungrouped SNP is left

If more than one candidate remains, then the $\left\lceil \#{\mathcal {T}}/2\right\rceil$-th SNP becomes the representative of group *b*. A next round of iteration is started using ${\mathcal {S}}_{b+1}={\mathcal {S}}_b\backslash {\mathcal {C}}_c$ until ${\mathcal {S}}_{b+1}=\emptyset$. The number of groups only depends on the threshold *t*. Similar to [2], $t = 0.8$ is a suitable value. In an extreme case (with *t* approaching 1), each SNP builds a single group, yielding a complexity of this algorithm of ${\mathcal {O}}(p^2)$, with *p* the total number of SNPs. This approach is implemented as function tagSNP in the R package hscovar. A graphical representation of this algorithm is shown in Fig. 1.

### Evaluation

It is an obvious choice to compare the family approach with a population-LD approach. Such an approach requires the population frequency of the different haplotypes ($f_{\text {A-A}}$, $f_{\text {A-B}}$, $f_{\text {B-A}}$, $f_{\text {B-B}}$) from which LD between markers is computed in terms of $r^2$ according to [5]. For any marker pair *k*, *l* with allele frequencies $f_k=f_{\text {A-A}}+f_{\text {A-B}}$ and $f_l=f_{\text {A-A}}+f_{\text {B-A}}$, we have

$$\begin{aligned} r^2_{k,l}&= \dfrac{\left( f_{\text {A-A}}f_{\text {B-B}}-f_{\text {A-B}}f_{\text {B-A}}\right) ^2}{f_k(1-f_k)f_l(1-f_l)}. \end{aligned}$$

The LD matrix can be computed from progeny genotypes using the function ld from the R package snpStats version 1.38.0 [6]. This function undertakes phasing of genotypes using a maximum-likelihood approach [7]. Based on the LD matrix containing $r^2$, representative markers can be derived as described above.

Family and population-LD approach were compared using the Calinski–Harabasz (CH) index [8] which measures the cluster quality with respect to inter- and intra-cluster distances. The method with higher CH index performed better. For this, the function calinhara from the R package fpc version 2.2-9 was applied to the groups obtained; the quality referred to distances based on the genotype matrix centered within family and scaled (as described below). Furthermore, we present the pure number of groups and highlight those groups with group size of at least three. This also helped visualizing the location of corresponding representative markers.

Selecting a representative set of markers is a natural tool for dimension reduction prior to genomic evaluations in order to reduce the impact of multicollinearity among predictor variables. Representative SNPs capture cumulative effects of the corresponding LD blocks on trait expression. We investigated a SNP-BLUP approach, which is widely used in genomic evaluations (e.g., [9]), and thereby demonstrate one possible application of the suggested approach. Representative markers selected from the family or from the population-LD approach were employed as predictor variables in a regression model

$$\begin{aligned} y&= X\beta +e, \end{aligned}$$

with $y=(y_1,\ldots ,y_n)^\top$ the phenotype vector, $\beta =(\beta _1,\ldots ,\beta _\tau )^\top$ the vector of genetic effects captured by $\tau$ tagSNPs, the corresponding design matrix *X* with dimensions $n\times \tau$ including the genotype codes in terms of major allele counts. The columns of *X* and the vector *y* were centered within family and scaled to obtain an empirical variance of one. The residual errors were assumed to be independently and normally distributed. For convenience, no other effects were assumed. We used the R package asreml version 3.0 [10] to estimate the vector of regression coefficients as

$$\begin{aligned} {\widehat{\beta }}&= \left( X^\top X+\lambda I\right) ^{-1}X^\top y \end{aligned}$$

where the shrinkage parameter $\lambda$ was estimated via AI-REML. Significance of the *k*-th SNP effect was tested by a *t*-like test statistic as in [1],

$$\begin{aligned} T_k&= \dfrac{{\widehat{\beta }}_k}{SD({\widehat{\beta }}_k)}. \end{aligned}$$

Significance was reported if $T_k \ge q_{1-\alpha /2}$ or $T_k <q_{\alpha /2}$ using the $1-\alpha /2$ and $\alpha /2$ quantile of the standard normal distribution. The SNP-BLUP approach was evaluated in terms of sensitivity (i.e., true-positive rate) and specificity (i.e., $1-$ false-positive rate) over a range of type-I error $\alpha$. We additionally verified the impact of threshold $t\in \{0.5,0.6,0.7,0.8\}$ on grouping and its consequences on the performance of the SNP-BLUP approach.

### Data

The data sets used for studying dependencies between SNP markers differed in SNP density and family structure. They covered a range of mean inter-marker distances from 0.003 cM to 0.23 cM. The case study of mouse data was based on low-density genotypes of full-sib families; progeny and parents were genotyped. The case study of cattle data comprised medium-density genotypes of half-sib families with genotyped progeny only. Furthermore, medium-density SNP data were available for full-sib families in maize. High-density genotype data of half-sib families were generated in simulations. For evaluation, the SNP-BLUP approach was applied to simulated data only. Unless otherwise stated, computations were done using R version 4.0.3 [11] and $t=0.8$; all scripts are included as Additional files 2–6.

#### Mouse data

Genotype data of a heterogeneous stock of mice were available from https://wp.cs.ucl.ac.uk/outbredmice/heterogeneous-stock-mice/. We investigated chromosome 17 because it harbors the highly recombining MHC region which affects several immunological traits [12]. The chromosome data consisted of genotypes at 394 SNPs of 2002 individuals. After filtering for individual call rate $\ge 90\%$, 1998 genotyped individuals remained comprising 1759 progeny, 120 fathers and 119 mothers. In total, 138 full-sib families (family size ranged from 1 to 47) could be identified. The SNP call rate was $\ge 90\%$. All genotype data were phased with Beagle version 5.1 [13] and parental haplotypes were selected to set up the correlation matrix. Assuming a 1:1 relationship between physical (Build37 genome assembly) and genetic distance of adjacent markers, the genetic length was 91 cM. SNP alleles were coded in terms of the major allele in the given sample. The population-LD matrix was calculated from progeny genotypes.

#### Cattle data

Genotype data of Holstein cattle were available from RADAR https://dx.doi.org/10.22000/280. The data comprised 50K SNP-chip data of five half-sib families with $n=265$ progeny in total; the family size ranged from 32 to 106. A chromosome window containing 300 SNPs was selected from BTA1. Based on the physical ordering of markers according to the genome assembly ARS-UCD1.2, this region corresponded to 20.59–39.44 Mbp. The haplotypes of sires were imputed from progeny genotypes using the R package hsphase version 2.0.2 [14]. Maternal LD and paternal recombination rates between SNP pairs were estimated according to Hampel et al. [15]. However, we used a 1:1 relationship between physical (Mbp) and genetic positions (cM) for convenience; the genetic length of this window was 19 cM. Sire haplotypes and maternal LD were also part of the RADAR data set. SNP alleles were coded in terms of the major maternal allele among progeny.

#### Maize data

Raw marker data were available from NCBI GEO database under Accession Number GSE50558, accompanied with physical coordinates corresponding to the genome assembly B73. The data set contained two maize panels, Flint and Dent, for which about 50K SNPs have been assessed in order to estimate recombination activity in different maize populations [16]. We arbitrarily chose the Flint panel and chromosome 2 for further analysis. In this panel, 13 full-sib families have been obtained by crossing an inbred “central” line and several inbred “founder” lines. Double haploid (DH) lines have been derived from the F1 plants. This procedure allowed for studying maternal meioses only. A cM:Mbp ratio of 0.80 was reported for Flint [16]; the genetic length of chromosome 2 was approximately 188 cM. SNP genotypes of DH progeny being heterozygous were set to missing value. After filtering the data for SNP and individual call rate $\ge 90\%$, $n=1248$ out of 1262 DH progeny and 1447 out of 2030 SNPs remained. Rarely missing marker information of DH progeny were imputed by sampling the homozygous genotypes according to their frequencies. Afterwards loci with minor allele frequency less than 5% were discarded, yielding 956 SNPs. As haplotypes of DH progeny were given with certainty, the population-LD matrix was set up directly using the squared Spearman correlation between SNPs based on haploid data. The family approach solely considered the female part of the covariance between SNPs. The haplotypes of F1 individuals were inferred from the marker data of inbred lines. SNP alleles were coded according to central-line origin.

#### Simulated data

The simulation study resembled the population structure of a dairy cattle population. The setup of simulation design is fully described in [1]. Briefly, we considered $N=1,5,10$ sires of half siblings. The overall number of progeny was $n=1000$ equally partitioned into half-sib families. Quantitative traits were simulated which were influenced by 2 and 5 QTLs with equal effect sizes. QTLs contributed 30% to the trait variation (i.e., heritability 0.3). In total, 300 SNPs were simulated on a chunk of DNA with 1 cM length. The data were generated using the R package AlphaSimR version 0.13.0 [17]. SNP alleles were recoded in terms of the major allele in the founder population. The simulation was repeated 100 times. For assessing the SNP-BLUP approach, a window of 0.05 cM to both sides of a simulated QTL was accepted as true-positive result.

## Results

### Case studies

**Fig. 2:**
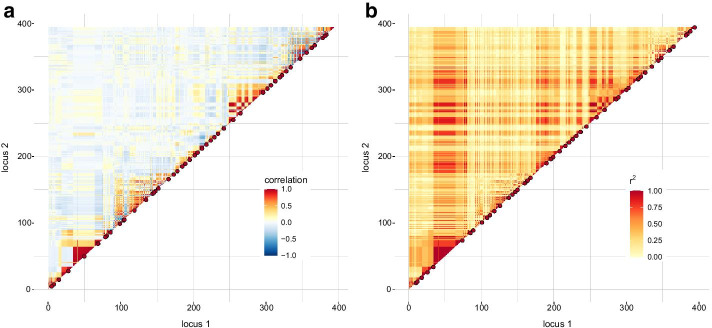
Correlation (**a**) and LD matrix (**b**) for mouse data on chromosome 17. The red dots highlight representative SNPs of groups with at least three SNPs. In total, 394 SNPs were considered for the correlation and population-LD matrix

For the mouse data consisting of 138 genotyped full-sib families, the population-LD approach exposed a wide region (13.82–21.13 Mbp) that had a strong association with the entire chromosome 17 shown as a red band in Fig. 2b. With the family approach, this region revealed only high positive interdependence with almost no impact on the remaining chromosome, see Fig. 2a. Moreover, a highly fragmented region appeared in the range of 27.59–45.88 Mbp that overlaps MHC regions 1 and 2 (https://www.ncbi.nlm.nih.gov/assembly/GCF_000001635.18/). This was caused by high variation of parental haplotypes. Though the total number of groups was smaller with the family approach than with the population-LD approach (83 vs. 98), the number of large groups, i.e., with group size $\ge 3$, was almost equal (49 vs. 51), see Table 1. The CH index was about two times higher with the family approach. Figure 2 shows that tagSNPs of groups containing at least three markers were distributed more uniformly over the chromosome with the family approach than with the population-LD approach. The median of distances between all tagSNPs was 0.70 cM and 0.52 cM in the family and population-LD approach, respectively.

**Table 1:** Number of groups, number of groups with at least three SNPs and Calinski–Harabasz index (CH)

	Families	QTLs	Family approach				Population-LD approach			
			SNPs	Groups	≥ 3	CH	SNPs	Groups	≥ 3	CH
Simulation	10	2	283	59	10	80.7	300	21	9	40.0
	10	5	282	61	10	75.0	300	17	9	38.9
	5	2	282	61	10	71.9	300	29	8	32.6
	5	5	281	64	10	85.6	300	24	9	35.0
	1	2	281	59	9	61.4	300	56	6	20.5
	1	5	282	59	9	83.0	300	49	7	25.0
Mouse	138		394	83	49	38.8	394	98	51	20.6
Cattle	5		237	172	11	2.9	300	210	11	2.4
Maize	13		953	426	93	10.4	956	576	70	21.9

Number of SNPs corresponds to the method applied (family or population LD). Average values of 100 repetitions are presented for simulated data and $t=0.8$. Computing time for grouping markers was up to 0.2 s except for the maize data which required 2 s

**Fig. 3:**
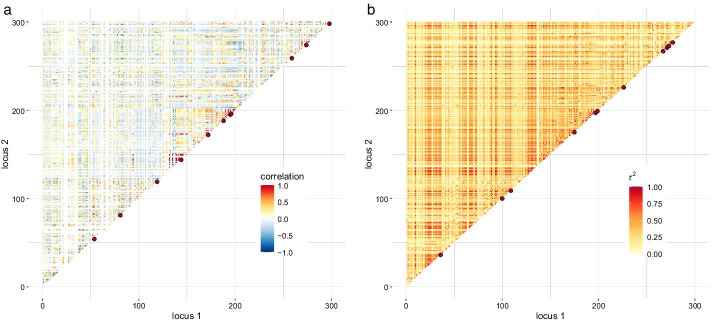
Correlation (**a**) and LD matrix (**b**) for cattle data in a target region of chromosome 1:20.6–39.4 Mbp. The red dots highlight representative SNPs of groups with at least three SNPs. In total, 300 SNPs were considered. The correlation matrix was set up at 239 SNPs and the population-LD matrix at 300 SNPs; missing values are filled in white color

For the cattle data consisting of five half-sib families, 239 SNPs were taken into account in the family approach. At 61 out of 300 SNPs, all sires were homozygous for the major allele, leading to zeros on the diagonal of the paternal covariance part. Thus, these loci were discarded when setting up the correlation matrix *R*. A clear distinction of regions with particularly high interdependence was not possible for any of the approaches (Fig. 3). In total, 11 groups with size $\ge 3$ were found with both the family and population-LD approach (Table 1) but the representative SNPs of these groups were distributed more evenly over the chromosome window based on the family approach. The median of distances between all tagSNPs was 0.08 cM and 0.07 cM in the family and population-LD approach, respectively. When only those SNPs were used in the population-LD approach that were considered in the family approach, the outcome of grouping differed: 239 SNPs were binned into 194 groups (CH index 6.2); 8 out of them had group size of at least three SNPs vs. 300 SNPs were binned into 210 groups (CH index 2.4); 11 groups with group size of at least three SNPs.

**Fig. 4:**
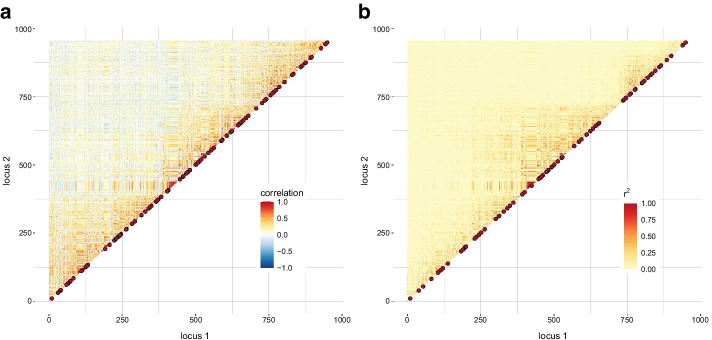
Correlation (**a**) and LD matrix (**b**) for maize data on chromosome 2. The red dots highlight representative SNPs of groups with at least three SNPs. In total, 956 SNPs were considered. The correlation matrix was set up at 953 SNPs and the population-LD matrix at 956 SNPs; missing values are filled in white color

The correlation matrix corresponding to 13 full-sib families in maize is shown in Fig. 4. In three out of 956 SNPs, maternal SNP alleles of F1 plants were missing; these loci were discarded in the family approach. In total, 953 SNPs have been binned into 426 groups based on the correlation matrix but the CH index was about 50% lower than with the population-LD approach where 956 SNPs were grouped into 576 bins (Table 1). With both approaches, tagSNPs corresponding to the bins of at least three SNPs were similarly distributed over the chromosome (family approach: 93 bins, population-LD approach: 70 bins) except a gap between SNP index 650 and 750 which was better covered with tagSNPs from the family approach. The median distance between all representative SNPs was 0.29 cM with the family and 0.16 cM with the population-LD approach. With both methods, a block of strong (positive) association among SNPs appeared in the region of 85.04 to 95.31 Mbp which is in the vicinity of the functional centromere [18]. Two F1 plants were the driving factor: they were completely heterozygous in this window.

### Simulation study

The average number of groups over all repetitions, and groups with at least three SNPs are listed in Table 1; results are given for $t=0.8$ and varying number of half-sib families and QTLs. Grouping of markers appeared rather robust based on the family approach, about 60 groups have been built, but the number of groups strongly varied with the population-LD approach suggesting a dependence on the number of families. Few families needed more groups. Note that the number of rows in *X* was fixed ($n=1000$). The number of groups containing at least three markers was rather constant between methods and for different family sizes and QTLs. The CH index was at least two times larger with the family approach than with the population-LD approach. The population-LD approach becomes competitive with decreasing threshold *t* and performed better than the family approach with respect to the CH index when $t\le 0.6$ (see Additional file 7).

**Fig. 5:**
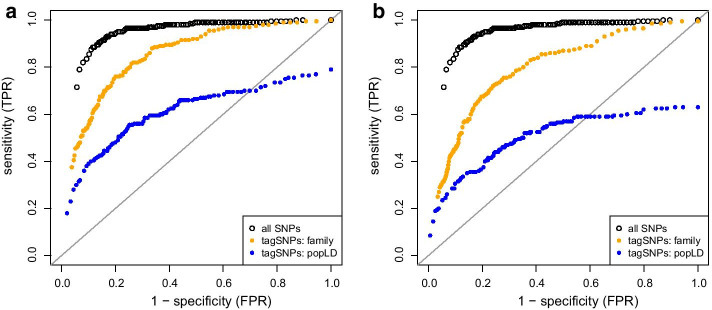
Sensitivity and specificity of testing SNP effects depending on threshold $t=0.8$ (**a**) and $t=0.5$ (**b**). ROC curves are based on 100 repeated simulations of genotypes and phenotypes in $N=1$ half-sib family with 1000 progeny (two QTL signals, heritability 0.3)

A SNP-BLUP approach was applied to simulated data in order to evaluate sensitivity and specificity if only tagSNPs were used as predictor variables in linear regression. As an example, results based on one half-sib family and two simulated QTLs are shown as ROC curve in Fig. 5; the number of groups was almost equal for this scenario. As expected, considering all SNPs simultaneously yielded highest accuracy in terms of true-positive and false-positive rate. However, if the aim was to use filtered data in SNP-BLUP, then tagSNPs obtained from the family approach was the second best choice. Or in other words, using only one fifth of available genotypic information led to almost the same accuracy of genome-based association studies as using all genotypic information. The choice of *t* had no influence on which method performed best, see Fig. 5a for $t=0.8$ and Fig. 5b for $t=0.5$. ROC curves looked very similar for all investigated scenarios of simulation though the number of groups obtained from the population-LD approach increased with decreasing number of families. Hence, a direct relationship between number of groups and sensitivity/specificity seems not to exist.

## Discussion

We have shown applicability of the suggested software tool to empirical data. Especially for the mouse data consisting of many genotyped full-sib families, the correlation matrix gave a clear representation of genomic regions with high or low interdependence. In contrast to a population-LD approach, which did not account for family stratification, it was also possible to identify regions with positive or negative relationship. Spurious dependencies with the population-LD approach disappeared with the family approach. Also Carlson et al. [2] reported that population stratification may generate artifactual LD and hence makes an LD-selection algorithm sensitive.

Instead of selecting representative markers for genome-based evaluations, employing the grouping structure itself can be a beneficial option. For instance, the group assignment derived from the family approach can directly be considered in a group lasso approach [3, 19]. Then the effects of markers in a group of highly dependent markers will jointly be shrunk towards zero or enlarged with respect to the relevance of this group for trait expression. Additional sparsity within group can be achieved with a sparse-group lasso approach [20]. Grouped approaches shall be investigated in more detail in future because they hold potential to cope with high multicollinearity. Possible benefits will likely depend on characteristics of the sample, such as the number of families, SNP density, and population-genetic parameters, e.g., heritability and heterozygosity.

In future research, the functionality of our package should be extended by grouping methods based on LD blocks which can optionally put restrictions to the physical distance between SNPs (similar to [21]). Other options for selecting tagSNPs (e.g., depending on allele frequency; [22]) will be verified.

## Conclusions

The extent of dependence among genomic markers is affected by the underlying population structure. Representative markers can be selected more efficiently if the corresponding matrix of pairwise dependencies takes this structure into account. The correlation matrix for half- or full-sib families highlights regions of high dependence between markers more precisely than the population-LD matrix. Additionally, it reveals regions of positive or negative association among markers.

We contributed a new function tagSNP to the R package hscovar which is suited to samples from livestock and crop populations with typical family stratification. The covariance matrix can be set up in a piecewise manner, either separately for each chromosome or based on other meaningful information. The resulting grouping structure can be exploited in genome-based evaluations to handle the problem of high multicollinearity between markers.

## Supplementary Information

**Additional file 1.** Given the population structure, half-sib families or full-sib families, the covariance matrix is analytically retrieved. Its computation using the R package hscovar is shown.

**Additional file 2.** Raw mouse data are processed and the matrix of correlation between markers is derived.

**Additional file 3.** Cattle data are processed and the matrix of correlation between markers is derived.

**Additional file 4.** Raw maize data are processed and the matrix of correlation between markers is derived.

**Additional file 5.** With this script, genotype and phenotype data of half-sib families are simulated and a genome-based association analysis is carried out.

**Additional file 6.** Plots of correlation matrices and population-LD matrices are produced based on results with Additional files 2–5.

**Additional file 7.** Number of groups, number of groups with at least three SNPs and Calinski-Harabasz index for different simulation scenarios and varying threshold.

## Data Availability

All R scripts used are provided in Additional files 2–6. The R package hscovar version 0.4.0 is available at CRAN. The cattle data are accessible through RADAR https://dx.doi.org/10.22000/280. The mouse data are obtainable from https://wp.cs.ucl.ac.uk/outbredmice/heterogeneous-stock-mice/. Maize data are available at NCBI GEO under Accession Number GSE50558.

## Abbreviations

AI-REML: Average information restricted maximum likelihood
BLUP: Best linear unbiased prediction
BTA: Bos taurus autosome
cM: CentiMorgan
DNA: Deoxyribonucleic acid
FPR: False positive rate
GWAS: Genomewide association study
LD: Linkage disequilibrium
Mbp: Mega base pairs
MHC: Major histocompatibility complex
QTL: Quantitative trait locus
ROC: Receiver operating characteristic
SNP: Single nucleotide polymorphism
TPR: True positive rate
