# Supplementary material for: Grouping of genomic markers in populations with family structure
Source: BMC Bioinformatics. 2021 Feb 19;22:79. doi: 10.1186/s12859-021-04010-0 (PMC7893918; doi:10.1186/s12859-021-04010-0)
Supplement: Supplementary file 1 — Additional file 1. Given the population structure, half-sib families or full-sib families, the covariance matrix is analytically retrieved. Its computation using the R package hscovar is shown. [file 12859_2021_4010_MOESM1_ESM.pdf]

Supplemental material to "Grouping of genomic markers in populations with family structure" by Wittenburg, Doschoris, Klosa (BMC Bioinformatics)

Family-based approach to the covariance between SNPs

The covariance between SNP pairs can be determined with respect to the family structure in a population. Its derivation requires phased genotypes of the common parent in case of half-sib families, and of all parents in case of full-sib families.

The design matrix X contains the genotype codes: $X_{j,k} \in \{1, 0, -1\}$ for progeny $j = 1, \ldots, n$ at SNP markers $k = 1, \ldots, q$. Homozygous genotypes A/A and B/B are coded as 1 and -1, respectively, and the heterozygous genotype A/B is indicated as 0. A genotype can be separated into independently inherited paternal (s) and maternal (m) SNP alleles: $X_{j,k} = X_{j,k,s} + X_{j,k,m}$, where $X_{j,k,s}$ and $X_{j,k,m}$ take a value of $\frac{1}{2}$ if the A allele was inherited and $-\frac{1}{2}$ otherwise.

The matrix $K = \{K_{k,l}\}$ of covariance between marker pairs k, l is set up according to Wittenburg et al. (2020). Key is the separation of the covariance $K_{k,l}$ into paternal ($D^{♂}_{k,l}$) and maternal contribution ($D^{♀}_{k,l}$) as $K_{k,l} = D^{♂}_{k,l} + D^{♀}_{k,l}$ because parental gametes are transmitted independently from parent to progeny (Bonk et al., 2016).

Half-sib families

Assuming that progeny have a common father and individual mothers, the maternal contribution is derived as linkage disequilibrium (LD) on maternal gametes: $D^{♀}_{k,l} = f_{\text{A-A}} f_{\text{B-B}} - f_{\text{A-B}} f_{\text{B-A}}$ with corresponding maternal haplotype frequencies at marker pair k, l. The paternal contribution is a function of the recombination rate between SNPs and depends on the haplotypes of each father. In case of several paternal half-sib families, the paternal covariance term $D^{♂}_{k,l}$ is obtained as the weighted average over covariance terms of individual fathers considering individual family sizes.

Let $\mathcal{M}$ ($\mathcal{F}$) denote the set of male (female) parents. Hence, assuming N families with sizes n_p ($p = 1, \ldots, N$) and $w_p = n_p/n$, it is

$$\begin{aligned} D^{♂}_{k,l} &= \sum_{p \in \mathcal{M}} w_p D_{k,l,p} + \sum_{p \in \mathcal{M}} w_p E(X_{j,k,p}) E(X_{j,l,p}) \\ &\quad - \sum_{p \in \mathcal{M}} w_p E(X_{j,k,p}) \sum_{p \in \mathcal{M}} w_p E(X_{j,l,p}) \,. \end{aligned} \tag{1}$$

This formula requires the covariance $D_{k,l,p}$ between SNPs on gametes of parent p,

$$D_{k,l,p} = \begin{cases} \frac{1}{4}(1 - 2\theta_{k,l}) \,, & \text{for parent } p \text{ with haplotypes A-A and B-B} \\ -\frac{1}{4}(1 - 2\theta_{k,l}) \,, & \text{for parent } p \text{ with haplotypes A-B and B-A} \\ 0 \,, & \text{else} \,, \end{cases}$$

the recombination rate $\theta_{k,l}$, which may be gender-specific or an average rate, and the expected value of a single allele transmitted from parent p,

$$E(X_{j,k,p}) = \begin{cases} \frac{1}{2} \,, & \text{for parent } p \text{ with genotype A/A} \\ 0 \,, & \text{for parent } p \text{ with genotype A/B} \\ -\frac{1}{2} \,, & \text{for parent } p \text{ with genotype B/B} \,. \end{cases}$$

We provide an implementation for setting up K in the R package `hscovar`. In paternal half-sib families, K is computed by

```
CovMat(linkMat = D, haploMat = haplotypes_of_fathers,
       nfam = family_size, pos = position_in_Morgan,
       map_fun = 'haldane')
```

Above, the $q \times q$ matrix D harbors the LD of gametes of the population the individual parents come from. It should not contain missing values. If maternal half-sib families are used, the roles of fathers and mothers are swapped. The following notations are used

`D`	$q \times q$ matrix of maternal LD
`D0`	$q \times q$ matrix of zeros
`haplotypes_of_fathers`	$2N_{♂} \times q$ matrix of haplotypes of fathers
`haplotypes_of_mothers`	$2N_{♀} \times q$ matrix of haplotypes of mothers
`family_size`	vector of family sizes $(n_1, \ldots, n_N)$
`position_in_Morgan`	list of vectors of genetic positions in Morgan units; one list entry for each chromosome or region
`map_fun`	genetic map function

The position of markers is provided in Morgan units but the user can choose a mapping function (`map_fun`) to convert genetic distances into recombination rates. So far, "haldane" (default) and "kosambi" are enabled. Haplotypes are coded with 0's and 1's reflecting the reference (B) and alternate (A) alleles, respectively, in two rows per individual. The number of parental haplotypes employed depends on the study design; $N_{♂}$ denotes the number of fathers and $N_{♀}$ the number of mothers. The vector `family_size` has matching length. If alleles are missing, those parents will be discarded at the corresponding pairs of SNPs when calculating the terms in Eqn. (1). Note that in `hscovar` version 0.4.0 missing alleles may be coded as any integer but not 0 and 1.

Full-sib families

An extension of the upper approach to full-sib families is straightforward. In such a case, also the maternal contribution to the covariance between SNPs can be described as a function of the recombination rate between SNPs depending on the haplotypes of each mother. Then the weighted average over covariance terms of individual mothers yields $D^{♀}_{k,l}$ as in Equation (1),

$$
\begin{aligned}
D^{♀}_{k,l} \;&=\; \sum_{p \in \mathcal{F}} w_p D_{k,l,p} + \sum_{p \in \mathcal{F}} w_p E(X_{j,k,p}) E(X_{j,l,p}) \\
&\quad - \sum_{p \in \mathcal{F}} w_p E(X_{j,k,p}) \sum_{p \in \mathcal{F}} w_p E(X_{j,l,p})\,.
\end{aligned}
$$

Applying the R package `hscovar` requires a detour as it was designed for half-sib families. Let D_0 denote an $q \times q$ matrix consisting of zeros. At first, $D^{♂}$ is determined by calling the function

```
CovMat(linkMat = D0, haploMat = haplotypes_of_fathers,
       nfam = family_size, pos = position_in_Morgan)
```

At second, $D^{♀}$ is computed by

```
CovMat(linkMat = D0, haploMat = haplotypes_of_mothers,
       nfam = family_size, pos = position_in_Morgan)
```

Eventually, $K = D^{\male} + D^{\female}$.

The correlation matrix can be achieved as `R = cov2cor(K)` but we also provide the option `corr = TRUE` when calling `CovMat` in order to derive correlations. SNPs that have variance larger than 10^{-6} are termed "valid", and the output is reduced to those SNPs.

References

S. Bonk, M. Reichelt, F. Teuscher, D. Segelke, and N. Reinsch. Mendelian sampling covariability of marker effects and genetic values. *Genetics Selection Evolution*, 48(1):36, 2016. doi: 10.1186/s12711-016-0214-0.

D. Wittenburg, S. Bonk, M. Doschoris, and H. Reyer. Design of experiments for fine-mapping quantitative trait loci in livestock populations. *BMC Genetics*, 21:66, 2020. doi: 10.1186/s12863-020-00871-1.
