# Supplementary material for: Grouping of genomic markers in populations with family structure
Source: BMC Bioinformatics. 2021 Feb 19;22:79. doi: 10.1186/s12859-021-04010-0 (PMC7893918; doi:10.1186/s12859-021-04010-0)
Supplement: Supplementary file 7 — Additional file 7. Number of groups, number of groups with at least three SNPs and Calinski-Harabasz index for different simulation scenarios and varying threshold. [file 12859_2021_4010_MOESM7_ESM.pdf]

Supplemental material to "Grouping of genomic markers in populations with family structure" by Wittenburg, Doschoris, Klosa (BMC Bioinformatics)

The influence of threshold t on grouping

Table 1: Number of groups, number of groups with at least three SNPs and Calinski-Harabasz index (CH). Number of SNPs corresponds to the method applied (family or population LD). Average values of 100 repetitions are presented for simulated data and $t = 0.7$.

			**Family approach**				**Population-LD approach**			
Data	Families	QTLs	SNPs	Groups	≥ 3	CH	SNPs	Groups	≥ 3	CH
Simulation	10	2	283	54	8	32.8	300	16	7	40.5
Simulation	10	5	282	56	9	35.5	300	12	8	41.0
Simulation	5	2	282	57	9	34.3	300	24	7	33.3
Simulation	5	5	281	59	9	35.4	300	19	7	35.5
Simulation	1	2	281	55	8	31.2	300	53	5	19.0
Simulation	1	5	282	54	8	40.3	300	46	5	25.8

Table 2: Number of groups, number of groups with at least three SNPs and Calinski-Harabasz index (CH). Number of SNPs corresponds to the method applied (family or population LD). Average values of 100 repetitions are presented for simulated data and $t = 0.6$.

			**Family approach**				**Population-LD approach**			
Data	Families	QTLs	SNPs	Groups	≥ 3	CH	SNPs	Groups	≥ 3	CH
Simulation	10	2	283	51	8	18.8	300	13	6	41.7
Simulation	10	5	282	52	8	16.1	300	10	6	38.4
Simulation	5	2	282	53	8	19.5	300	21	6	31.6
Simulation	5	5	281	55	8	14.5	300	16	6	36.5
Simulation	1	2	281	52	8	19.8	300	52	4	14.6
Simulation	1	5	282	51	8	27.6	300	44	4	22.6

Table 3: Number of groups, number of groups with at least three SNPs and Calinski-Harabasz index (CH). Number of SNPs corresponds to the method applied (family or population LD). Average values of 100 repetitions are presented for simulated data and $t = 0.5$.

			**Family approach**				**Population-LD approach**			
Data	Families	QTLs	SNPs	Groups	≥ 3	CH	SNPs	Groups	≥ 3	CH
Simulation	10	2	283	47	7	9.9	300	11	5	41.9
Simulation	10	5	282	49	8	10.2	300	7	5	38.8
Simulation	5	2	282	49	7	12.0	300	19	4	32.6
Simulation	5	5	281	51	8	8.9	300	14	5	38.2
Simulation	1	2	281	48	7	11.4	300	50	3	15.6
Simulation	1	5	282	48	7	21.4	300	43	3	24.2
